# Performance of the ROX index in predicting high flow nasal cannula failure in COVID-19 patients: a systematic review and meta-analysis

**DOI:** 10.1186/s13054-023-04567-7

**Published:** 2023-08-21

**Authors:** Chun En Yau, Dawn Yi Xin Lee, Adithi Vasudevan, Ken Junyang Goh, Evelyn Wong, Andrew Fu Wah Ho, Daniel Yan Zheng Lim

**Affiliations:** 1 Yong Loo Lin School of Medicine, National University of Singapore, Singapore, Singapore; 2 School of Medicine, Dentistry and Nursing, University of Glasgow, Glasgow, UK; 3 Department of Respiratory and Critical Care Medicine, Singapore General Hospital, Singapore, Singapore; 4 Department of Emergency Medicine, Singapore General Hospital, Singapore, Singapore; 5 Pre-Hospital and Emergency Research Centre, Duke-National University of Singapore Medical School, Singapore, Singapore; 6 Centre for Population Health Research and Implementation, SingHealth Regional Health System, Singapore, Singapore; 7 Saw Swee Hock School of Public Health, National University of Singapore, Singapore, Singapore; 8 Department of Gastroenterology and Hepatology, Singapore General Hospital, Singapore, Singapore

**Keywords:** ROX index, COVID-19, High flow nasal cannula, Systematic review, Meta-analysis, Respiratory failure

## Abstract

COVID-19 patients with acute hypoxemic respiratory failure (AHRF) benefit from high flow nasal cannula (HFNC) oxygen therapy. However, delays in initiating invasive ventilation after HFNC failure are associated with poorer outcomes. The respiratory oxygenation (ROX) index, combining SpO_2_/FiO_2_ and respiratory rate, can predict HFNC failure. This meta-analysis evaluated the optimal ROX index cut-offs in predicting HFNC failure among COVID-19 patients at different measurement timings and clinical settings. Three databases were searched for eligible papers. From each study, we reconstructed the confusion matrices at different cut-offs, fitted linear mixed models to estimate the ROX index distribution function, and derived the area under the summary receiver operator characteristic curve (sAUC) and optimal cut-offs to predict HFNC failure. 24 studies containing 4790 patients were included. Overall sAUC was 0.771 (95% CI: 0.666–0.847) (optimal cut-off: 5.23, sensitivity: 0.732, specificity: 0.690). The cut-off values to achieve 80%, 90% sensitivity, 80%, 90% specificity were 5.70, 6.69, 4.45, 3.37, respectively. We stratified the analysis by ROX measurement time and estimated optimal cut-offs and cut-offs to achieve 80% sensitivity and specificity. For 2–6 h and 6–12 h post-HFNC initiation, we propose the use of 80% specific cut-offs to rule in HFNC failure of < 5.33 and < 3.69, respectively. For 12–24 h post-HFNC initiation, we propose the use of the 80% sensitive cut-off of > 6.07 to rule out HFNC failure. Our analysis confirms the overall utility of the ROX index in risk stratification of COVID-19 patients with AHRF receiving HFNC and provides potentially useful cut-offs for different times from HFNC initiation.

**Supplementary Information:**

The online version contains supplementary material available at 10.1186/s13054-023-04567-7.

## Introduction

The COVID-19 pandemic is an unprecedented global health crisis, with more than 651 million infections and more than 6.6 million deaths [1]. A serious complication and common cause of death in patients with COVID-19 infection is acute hypoxemic respiratory failure (AHRF), which occurs in 15–30% of COVID-19 patients [2]. This can be managed by high flow nasal cannula (HFNC) oxygen therapy [3], a non-invasive method of oxygen supplementation, using a large-bore nasal cannula to administer up to 100% FiO_2_ at a high flow of 60 L/min [4, 5]. This intervention creates positive airway pressure [6] and lessens the anatomical dead space and the work of breathing [7]. The use of warmed (31–37 °C) [8], humidified oxygen protects the mucosal lining, allows oxygenation with lower transpulmonary driving pressure, and facilitates secretion clearance [9]. HFNC can be used to spare patients from invasive mechanical ventilation [10].

However, HFNC use can delay endotracheal intubation, and such delays are associated with longer invasive mechanical ventilation and a poorer prognosis [11]. In particular, COVID-19 patients have a high failure rate of non-invasive treatment (i.e., worsening in severity so as to require intubation and invasive ventilation) [12]. Hence, prognostic tools to predict HFNC failure have high clinical relevance, to discriminate in a timely manner the patients who are poor candidates for HFNC continuation, from those who can be safely spared from invasive mechanical ventilation. Current risk stratification tools for HFNC failure include respiratory parameters—the oxygen saturation to fraction of inspired oxygen ratio (SpO_2_/FiO_2_), respiratory rate (RR), partial pressure of carbon dioxide (PaCO_2_), and partial pressure of oxygen (PaO_2_) [13, 14]. However, these parameters in isolation are unable to identify the need for intubation reliably [15].

The respiratory oxygenation (ROX) index is a prognostic index that has gained popularity during the COVID-19 pandemic, and was specifically developed to prognosticate HFNC failure in patients with pneumonia and AHRF [16–18]. It combines SpO_2_/FiO_2_ and respiratory rate (RR) using the formula $\left( {\frac{{{{{\text{SpO}}_{2} } \mathord{\left/ {\vphantom {{{\text{SpO}}_{2} } {{\text{FiO}}_{2} }}} \right. \kern-0pt} {{\text{FiO}}_{2} }}}}{{{\text{RR}}}}} \right)$ [15], and can be easily done at the bedside [19]. The original description of the ROX index by Roca et al. measured the ROX at 2, 6, and 12 h after HFNC initiation. The proposed cut-offs were ROX > 4.88 to predict HFNC success at 2, 6, and 12 h, and ROX < 3.85 after 12 h to predict HFNC failure. A ROX of 3.85–4.88 was described as an indeterminate range, and the authors suggested that such patients should have the ROX reassessed at a later time point.

Multiple studies have validated the ROX index in recent years [20, 21]. However, as there are no universally accepted protocols for ROX use, studies on the ROX index have adopted different cut-offs and monitoring intervals. There is thus uncertainty [17, 21] over optimal cut-off values in general, and the specific cut-offs when ROX is measured at different times from HFNC initiation. We note a previous meta-analysis by Prakash et al. [19], which included all studies on ROX as a predictor for HFNC failure up till early 2021. Prakash et al. provided a simple dichotomisation of the ROX index into high versus low categories, with no specific analysis of time from ROX initiation. There are no present meta-analyses to determine the optimal ROX index cut-offs [8, 19, 22], at initiation of HFNC and at subsequent time points.

Hence, in this systematic review and meta-analysis, we aimed to describe the performance of the ROX index in predicting HFNC failure amongst COVID-19 patients at different time points from HFNC initiation. We further aimed to derive optimal cut-off values at various timings to guide the interpretation of ROX index in various clinical settings.

## Methods

### Search strategy

The Preferred Reporting Items for Systematic Reviews and Meta-Analyses (PRISMA) guidelines [23] informed the design and execution of this study. The protocol was registered on PROSPERO (CRD42023388254). Medline/PubMed, Embase and Cochrane Central Register of Controlled Trials (CENTRAL) were searched from inception to 20 December 2022 for eligible studies with keywords related to Covid-19 and ROX index. The search strategy was developed in collaboration with a medical librarian (Medical Library, National University of Singapore). The search strategy can be found in the Additional file 1. No language filters were applied. Two authors independently carried out the preliminary eligibility screening in a blinded fashion. The authors screened the titles and abstracts before retrieving and reviewing the full texts. Studies were included if they (1) included Covid-19 patients on HFNC and (2) utilised the ROX index. Reviews, commentaries, animal studies and case reports were excluded. A senior author resolved differences by discussion and consensus.

### Data extraction and selection criteria

From each study, two authors used a standardised data extraction sheet to extract information on the study period, country, population demographics independently. ROX cut-off values for each study, and the definition of HFNC failure or success were extracted. Since some studies used HFNC success as the outcome, while others used HFNC failure, we standardised HFNC failure as the outcome of interest. HFNC failure included the outcomes of intubation, escalation to mechanical ventilation (including non-invasive and invasive ventilation), and death.

Confusion matrices (a form of the 2 × 2 contingency table) were constructed for each study detailing the number of true positives (patients who scored below the ROX cut-off and experienced HFNC failure), true negatives (patients who scored above the ROX cut-off and experienced HFNC success), false positives (patients who scored below ROX cut-off and experienced HFNC success), and false negatives (patients who scored above the ROX cut-off and experienced HFNC failure).

### Statistical analysis

All analysis was done using RStudio (version 2021.9.1.372). Statistical analysis was conducted with meta (version 5.2–0) and diagmeta (version 0.5–0) packages. Using diagmeta which implements the approach outlined by Steinhauser et al. [24] and also previously successfully applied to other acute clinical research questions [25], various linear mixed models were fitted to estimate the distribution function of the ROX index within the included studies. For the linear mixed models that converged, we applied the restricted maximum likelihood criterion and the model that minimised this criterion was selected. Area under the summary receiver operator characteristic curve (sAUC) was derived for different individual time points and time windows, and the optimum cut-off values were calculated. The optimum cut-off value was the cut-off which maximised the weighted sum of sensitivity and specificity.

### Subgroup analysis

Analysis of the diagnostic accuracy of the ROX index was conducted in prespecified subgroups: average age of included patients, admission year of patients, corticosteroid usage. Sensitivity analysis was conducted by excluding conference abstracts and letters and including only journal articles. Further sensitivity analysis was conducted for the type of outcome—excluding studies that had progression to NIV or death as an outcome. Pairwise comparison of sAUC values between different subgroups was made in accordance with the methods outlined by Hanley et al. [26, 27].

### Risk of bias assessment

Two independent and blinded authors assessed studies for methodological quality, using the Quality Assessment of Prognostic Accuracy Studies (QUAPAS) tool for prognostic studies [28]. The QUAPAS tool assesses the quality of studies across five key domains: participant selection, index test, outcome, flow and timing, and analysis. Disagreements were resolved through discussion with a third author.

### Patient and public involvement

No patients or members of the public were directly involved in this research study.

## Results

### Article search and included studies

**Fig. 1:**
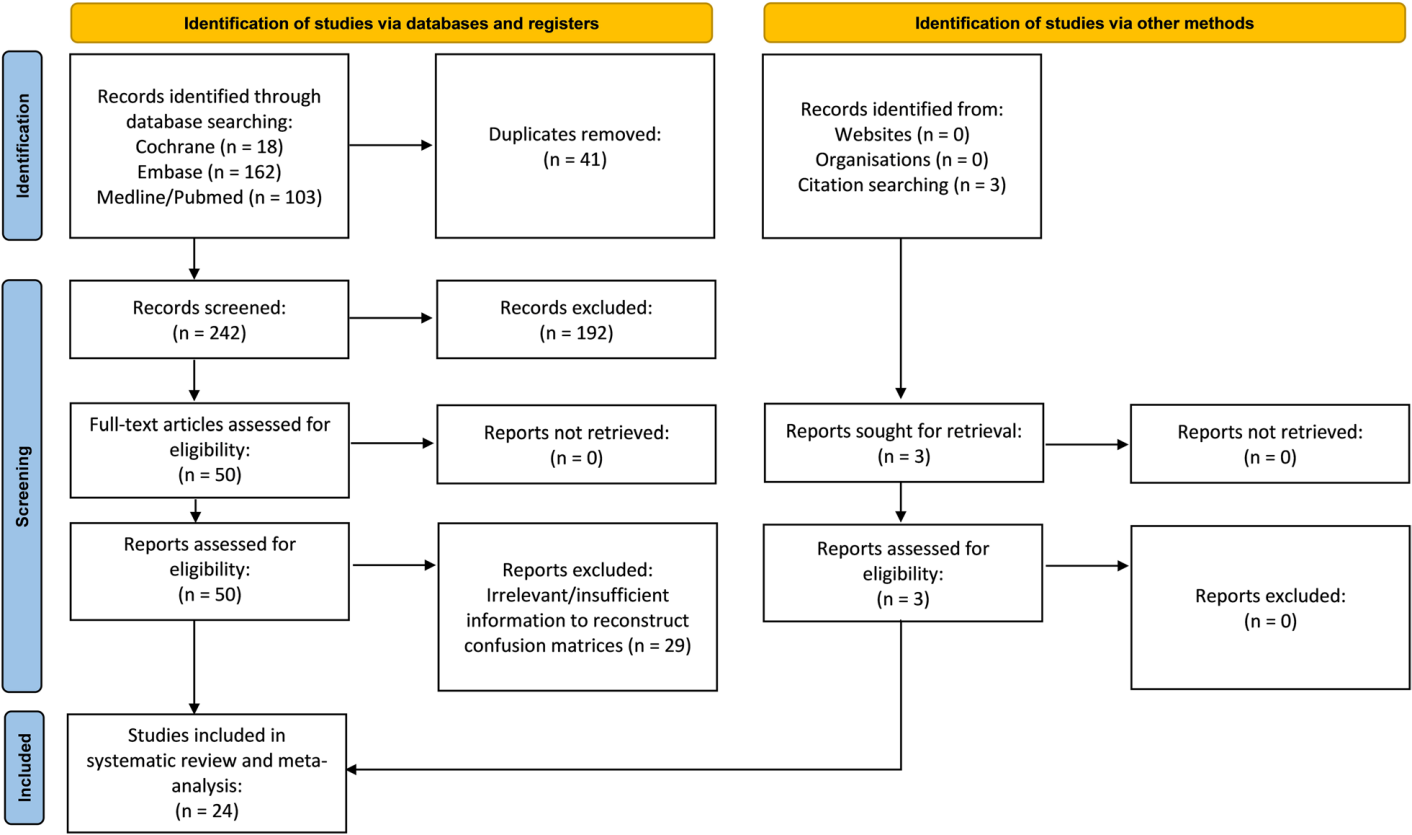
Preferred reporting items for systematic reviews and meta-analyses (PRISMA) flow chart

The search strategy identified 242 relevant studies (Fig. 1) after removal of duplicates. Fifty studies were included in the full-text review. Our final analysis included 24 [16, 21, 29–50] studies, comprising 4790 patients (Table 1). Of the 24 studies, nine were multicentre studies and 15 were single-centre studies.

**Table 1:** Characteristics of included studies

First author	Number of centres	Study type	Country of population	Year of publication	Study time period	Mean age	Time points/time windows	Cut-off(s)	Time from HFNC initiation to intubation	Definition of HFNC success/failure	Number of HFNC failures	Number of HFNC successes	Number of included patients
Hu	Multicentre	Retrospective	China	2020	2020	64	6 h	5.55	NR	HFNC withdrawal with improved oxygenation, not requiring NIV/IMV, discharged alive	40	65	105
Bruna	Single-centre	Prospective	Chile	2022	2020	63.3	12 h	6.80, 7.30	NR	Intubation, death	16	25	41
Talpos	Single-centre	Retrospective	Belgium	2022	2020	70	0-24 h	5.63	NR	IMV	31	21	52
Duan	Multicentre	Retrospective	China	2021	2020	67.4	1 h, 2 h, 4 h, 8 h, 12 h, 24 h	5.49, 5.63, 5.94, 6.25, 7.17, 7.93	Median 41 IQR (19–152) hours	NIV, IMV	29	37	66
Chandel	Multicentre	Retrospective	USA	2021	2020	57	2 h, 6 h, 12 h, 2-12 h	3, 3.41, 3.46, 3.67, 4.57	NR	HFNC successfully weaned	108	164	272
Kim	Multicentre	Retrospective	Korea	2022	2020 to 2021	70	1 h	8.54	NR	Intubation, IMV	70	63	133
Ferrer	Single-centre	Prospective	Spain	2021	2021	64.5	1 h, 6 h, 12 h, 24 h	5.25, 5.27, 5.35, 5.41	NR	IMV, NIV, death	47	38	85
Calligaro	Multicentre	Prospective	South Africa	2020	2020	51.3	6 h	2.2, 2.7, 3.7	Median 2 IQR (0.5–5) days	Intubation, death	156	137	293
Myers	Multicentre	Retrospective	USA	2022	2020	61.4	12 h	3.85, 4.88	Median 83.5 IQR (27.8–178.9) hours	IMV	585	1262	1847
Metwaly	Single-centre	NR	NR	2021	NR	NR	24 h	5.2	NR	HFNC weaned with improved oxygenation	15	60	75
Hamou	Single-centre	Retrospective	France	2022	2020 to 2021	67	6 h	1.96	NR	Intubation	78	36	114
Vega	Multicentre	Retrospective analysis of prospectively collected data	Argentina and Italy	2022	2020	NR	2 h, 6 h, 12 h, 24 h	5.1, 5.8, 5.99, 8.36	Median 2 IQR (1–3) days	IMV, death	35	85	120
Blez	Single-centre	Prospective	France	2020	2020	NR	0.5 h	3.8	NR	IMV within 7 days of HNFC	16	14	30
Zucman	Single-centre	Retrospective	France	2020	2020	55.3	0-4 h	5.37	NR	HFNC successfully weaned	39	21	60
Takeshita	Single-centre	Retrospective	Japan	2022	2021	57.9	NR	5.55	NR	IMV	14	22	36
Burnim	Multicentre	Retrospective	USA	2021	2020	63	5-12 h	3.85	NR	IMV	266	196	462
Fernando Valencia	Single-centre	Retrospective	Columbia	2021	2020	62.5	2 h	5.6	NR	Intubation, death due to COVID-19	152	93	245
Gaspic	Single-centre	Retrospective	Croatia	2021	2020 to 2021	65.7	NR	4.12	NR	In-hospital death	42	60	102
Kucuk	Single-centre	Retrospective	Turkey	2022	2020 to 2021	66.7	1 h	3.81	NR	IMV, death	52	33	85
Panadero	Single-centre	Retrospective	Spain	2020	2020	58.9	2-6 h	4.94	Median 2 IQR (1–4) days	Intubation	21	19	40
Aparicio	Single-centre	Retrospective analysis of prospectively collected data	Spain	2020	2020	60.8	12 h	5.57	NR	Not requiring NIV, IMV, ECMO; no death; within 28 days of HFNC	6	15	21
Tzouanatou	Single-centre	Prospective	Greece	2022	2021	57	6 h	4.79	Mean 4.48 days	Sustained severe hypoxemia (PaO2/FiO2 < 100) requiring IMV and ICU admission	23	46	69
Wojcik	Single-centre	Prospective	Poland	2021	2021	60.7	0-12 h	3.85, 4.88	Survivors: Median 1 day Non-survivors: Median 3 days	Not requiring intubation	63	50	113
Xu	Multicentre	Retrospective	China	2020	2020	63.2	0-4 h	5.31	NR	IMV within 7 days of HFNC initiation	147	177	324

*HFNC* high flow nasal cannula [used synonymously with *HFNO* (high-flow nasal oxygen), *HVNI* (high-velocity nasal insufflation)]; *IMV* invasive mechanical ventilation; *NIV* non-invasive ventilation; *ECMO* extracorporeal membrane oxygenation; *ICU* intensive care unit; *SD* standard deviation; *IQR* interquartile range; *NR* not reported

Studies came from all major global regions, including one multicontinental study involving two institutions from Europe and South America. Studies from single regions included one from Africa, five from Asia, eleven from Europe, three from North America, and two from South America. One did not report the location of the study.

Six were prospective studies, 15 were retrospective studies, and two were retrospective analyses of prospectively collected data. One study did not report on the study type. Seven studies were published in 2020, eight in 2021, and nine in 2022.

The mean age of included patients ranged from 51.3 to 70. ROX cut-offs investigated ranged from 1.96 to 8.36. The definitions of HFNC success or failure used by each study are shown in Table 1.

Most of the studies were evaluated to be moderate or high in bias (Additional file 1: Fig. S1).

### Overall performance of the ROX index

**Fig. 2:**
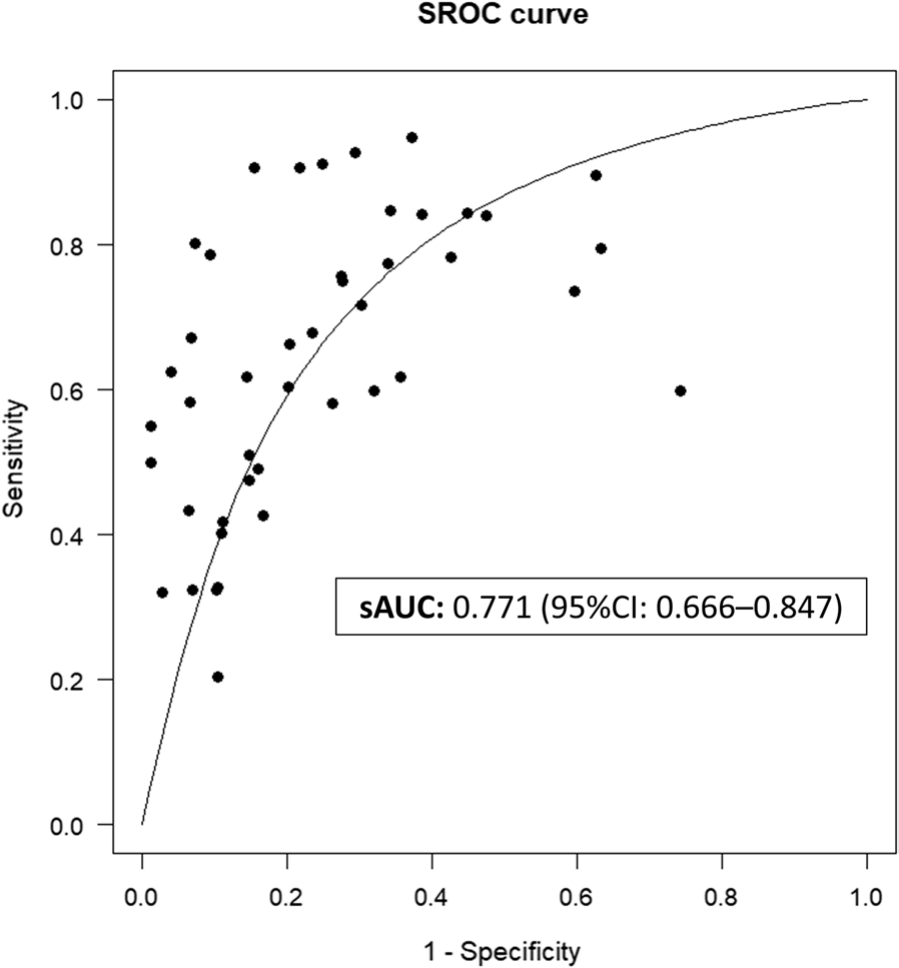
Overall summary receiver operator characteristic curve

Results from the 24 studies were pooled. ROX had an overall sAUC of 0.771 (95% CI: 0.666–0.847) (Fig. 2), and the overall optimal cut-off value of ROX was 5.23. At this cut-off, sensitivity was 0.732 (95% CI: 0.578–0.846) and specificity was 0.690 (95% CI: 0.539–0.809). The cut-off to achieve 80% sensitivity was 5.70, while the cut-off to achieve 80% specificity was 4.45. The cut-off to achieve 90% sensitivity was 6.69, while the cut-off to achieve 90% specificity was 3.37.

A sensitivity analysis was conducted by including only original journal articles (i.e. excluding letters and abstracts). In these 19 studies with 4535 patients, sAUC was 0.770 (95% CI: 0.656–0.849), and the optimal cut-off value was 5.27. At this cut-off, sensitivity was 0.741 (95% CI: 0.563–0.864) and specificity was 0.681 (0.515–0.811). No significant difference in sAUC was found (two-tailed *p*-value: 0.921). Another sensitivity analysis was conducted by including original journal articles and letters (i.e. excluding abstracts). In these 21 studies with 4625 patients, sAUC was 0.764 (95% CI: 0.648–0.845), and the optimal cut-off value was 5.10. At this cut-off, sensitivity was 0.722 (95% CI: 0.552–0.846) and specificity was 0.688 (0.526–0.814). No significant difference in sAUC was found (two-tailed *p*-value: 0.488).

As a further sensitivity analysis, we analysed the overall optimal cut-offs with the exclusion of Hamou et al. [38] (with the lowest cut-off), and Kim et al. [33] (with the highest cut-off), removed from the analysis separately. Removing Hamou et al., sAUC was 0.766 (95% CI: 0.661–0.841); removing Kim et al., sAUC was 0.779 (95% CI: 0.688–0.848); removing both, sAUC was 0.774 (95% CI: 0.684–0.842). There was no significant difference from the overall sAUC in these three sensitivity analyses (two-tailed *p*-value 0.620, 0.423 and 0.767, respectively).

### Diagnostic accuracy of the ROX index measured within specific time windows

**Fig. 3:**
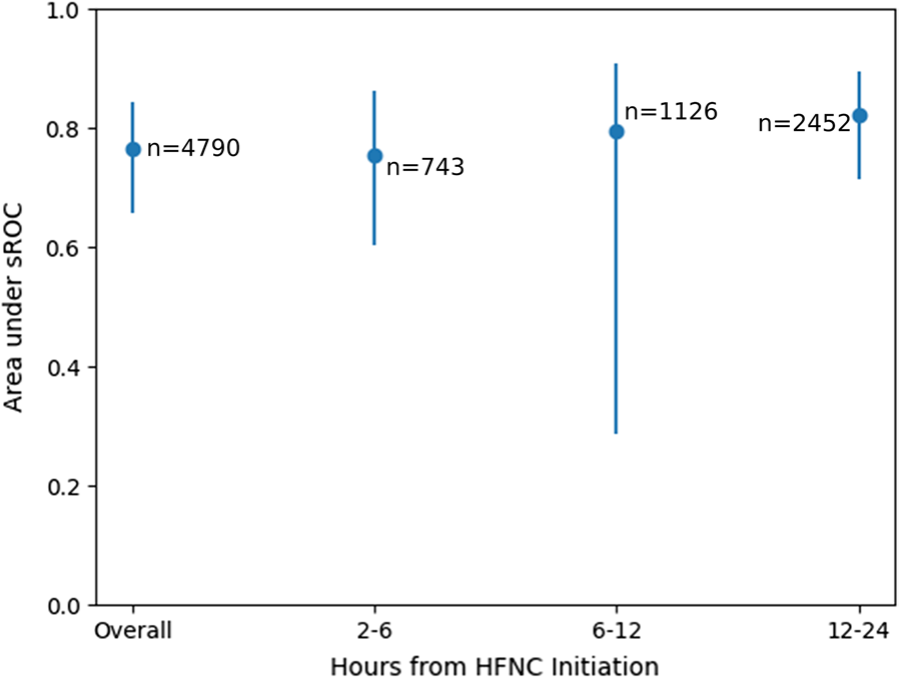
Area under summary receiver operator characteristic curve at various time windows with 95% confidence intervals

In 5 studies which used ROX measured ≥ 2 h but < 6 h of initiation of HFNC, sAUC was 0.754 (95% CI: 0.604–0.863) (Fig. 3), and the optimal cut-off was 5.71. At this cut-off, sensitivity and specificity were 0.635 (95% CI: 0.411–0.813) and 0.769 (95% CI: 0.465–0.927), respectively (Table 2).

**Table 2:** Diagnostic accuracy at various time windows

Time window	Number of studies	Number of patients	Number of cut-offs	sAUC	Optimal cut-off	Sensitivity at optimal cut-off	Specificity at optimal cut-off	Cut-off for 80% sensitivity	Cut-off for 80% specificity	*p*-value*
≥ 2 h, < 6 h	5	743	6	0.754 (95% CI: 0.604–0.863)	5.71	0.635 (95% CI: 0.411–0.813)	0.769 (95% CI: 0.465–0.927)	7.96	5.33	NA
≥ 6 h, < 12 h	8	1126	10	0.795 (95% CI: 0.287–0.908)	6.50	0.835 (95% CI: 0.389–0.976)	0.659 (95% CI: 0.192–0.940)	5.95	3.69	0.069
≥ 12 h, < 24 h	7	2452	10	0.821 (95% CI: 0.713–0.894)	5.78	0.749 (95% CI: 0.575–0.868)	0.759 (95% CI: 0.511–0.905)	6.07	5.55	0.001

*sAUC* area under the summary receiver operator characteristic curve; *CI* confidence interval; *NA* not applicable

*p-value for difference, using ≥ 2 h, < 6 h window as the base comparator

In 8 studies which used ROX measured ≥ 6 h but < 12 h of initiation of HFNC, sAUC was 0.795 (95% CI: 0.287–0.908) (Fig. 3), and the optimal cut-off was 6.50. At this cut-off, sensitivity and specificity were 0.835 (95% CI: 0.389–0.976) and 0.659 (95% CI: 0.192–0.940), respectively (Table 2).

In 7 studies which used ROX measured ≥ 12 h but < 24 h of initiation of HFNC, sAUC was 0.821 (95% CI: 0.713–0.894) (Fig. 3), and the optimal cut-off was 5.78. At this cut-off, sensitivity and specificity were 0.749 (95% CI: 0.575–0.868) and 0.759 (95% CI: 0.511–0.905), respectively (Table 2).

No analysis of the ROX was done for < 2 h from ROX initiation as there were only 5 studies with 5 cut-offs, insufficient to fit the linear mixed models for the ROX index distribution in this time window.

### Diagnostic accuracy of the ROX index in clinically important subgroups

#### Elderly patients

Twenty one studies included data on the average age of patients. No significant difference in sAUC value was found between studies where the mean age was less than 65 and those where the mean age was more than or equal to 65 (two-tailed *p*-value: 0.067).

#### Use of corticosteroids

In five studies where more than 75% of patients received corticosteroid treatment, sAUC of ROX was 0.711 (95% CI: 0.293–0.883). In three studies where the proportion of patients who received corticosteroid treatment was less than or equal to 75%, sAUC of ROX was 0.721 (95% CI: 0.005–0.997). sAUC values were not significantly different between the two groups (two-tailed *p*-value: 0.696).

#### Admission year of patients

Fifteen studies included patients admitted in 2020 only, while four studies included patients admitted in 2021 only. For this sub-analysis, we did not include four studies which had included patients across both 2020–2021, and one study that did not state the year of admission. sAUC value in the group admitted in 2021 was insignificantly different from that in the group admitted in 2020 (two-tailed *p*-value: 0.404).

The sAUC values and *p*-values for between subgroup differences are detailed in Table 3.

**Table 3:** Area under the summary receiver operator characteristic curve (sAUC) in clinically important subgroups

	Number of studies	Number of patients	Number of cut-offs	sAUC	*p*-value for between- subgroup difference
*Average age*					
< 65	15	4013	27	0.757 (95% CI: 0.646–0.837)	0.067
≥ 65	6	552	11	0.715 (95% CI: 0.439–0.877)	
*Proportion of patients on corticosteroid treatment*					
> 75%	5	755	8	0.711 (95% CI: 0.293–0.883)	0.696
≤ 75%	3	819	7	0.721 (95% CI: 0.005–0.997)	
*Admission year*					
2020	15	3978	31	0.750 (95% CI: 0.633–0.833)	0.404
2021	4	303	8	0.774 (95% CI: 0.085–0.980)	

*CI* confidence interval

#### Other subgroups

Three studies of the ROX index were performed in unvaccinated COVID-19 patients. The cut-offs in these few studies widely ranged from 1.96 to 5.63, and this can affect the quality of sAUC estimate. In particular, Hamou et al. used an unusually low cut-off of 1.96. Most studies did not report on the vaccination status of their included patients. There were no studies which made direct comparison of the ROX index between vaccinated and non-vaccinated patients. We decided not to perform further analysis of the ROX index based on vaccination status.

### Diagnostic accuracy of the ROX index for different outcomes

The definition of HFNC failure was heterogenous across different studies, with some studies including mortality as part of a composite outcome, and some including progression to NIV as an endpoint. We present the diagnostic accuracy of the ROX index stratified by the definition of HFNC failure in Table 4.

**Table 4:** Diagnostic accuracy stratified by definition of high flow nasal cannula failure

Nature of outcome	Number of studies	Number of patients	Number of cut-offs	sAUC	Optimal cut-off	Sensitivity at optimal cut-off	Specificity at optimal cut-off	Cut-off for 80% sensitivity	Cut-off for 80% specificity
Intubation, mechanical ventilation, non-invasive ventilation, or death	24	4790	44	0.771 (95% CI: 0.666–0.847)	5.23	0.732 (95% CI: 0.578–0.846)	0.690 (95% CI: 0.539–0.809)	5.70	4.45
Intubation or mechanical ventilation	13	3505	23	0.722 (95% CI: 0.535–0.845)	5.18	0.634 (95% CI: 0.506–0.745)	0.710 (95% CI: 0.566–0.821)	7.06	4.20
Intubation, mechanical ventilation, or death	9	1108	19	0.785 (95% CI: 0.588–0.891)	4.90	0.730 (95% CI: 0.436–0.904)	0.716 (95% CI: 0.422–0.897)	5.24	4.48

*sAUC* area under the summary receiver operator characteristic curve, *CI* confidence interval

## Discussion

HFNC is an important ventilation sparing therapy in COVID-19 patients. However, delayed intubation in HFNC has been shown to lead to increased mortality [11]. Use of the ROX index has allowed for objective assessment of HFNC failure risk, to allow early stratification of patients who can be safely continued on HFNC, from those who are likely to need invasive ventilation. Previous meta-analyses [8, 19] have dichotomised patients to an overall high or low ROX index, to handle different ROX cut-off values used in different studies. On the other hand, our up-to-date systematic review and meta-analysis of the ROX index in COVID-19 patients has included information on all cut-off values and time points described in the constituent studies. We have modelled the sROC of the ROX index in general, as well as at different time points. This has allowed us to estimate the optimal cut-off at each time point, as well as the clinically relevant 80% sensitivity and specificity cut-offs.

Overall, we found that the ROX index had a sAUC of 0.771. The optimal cut-off of the ROX index in general that maximised sensitivity and specificity for HFNC failure was 5.23 (sensitivity: 0.732, specificity: 0.690). This is higher than the cut-off proposed by Roca et al. [18] (who originally developed the ROX index), where a universal cut-off of ROX > 4.88 at 2, 6, and 12 h post-HFNC initiation was suggested to predict HFNC success. Our cut-off derived from analysis of the sAUC agrees more with subsequent studies such as Prakash et al. and Zhou et al. [19, 20], which have suggested an optimal ROX index cut-off of around 5. We suggest that if a general cut-off for the ROX index is sought, without regard for time from initiation, that 5.23 be used as it maximises both sensitivity and specificity. Alternatively, cut-off values of 5.70 and 4.45 are 80% and 80% sensitive and specific, respectively, while cut-off values of 6.69 and 3.37 are 90% sensitive and 90% specific, respectively, in determining HFNC failure without regard for time from initiation. These cut-offs could be used if a provider prefers higher sensitivity or specificity.

For most patients on HFNC therapy, the time from initiation is known, and the ROX can be calculated at different times from HFNC initiation. We provide a further meta-analysis of the performance of the ROX index at different time windows. Providers should be aware of the differences in ROX index performance at different time windows and note the ROX index performs better at time windows from 6 h onwards. We observed that the sAUC improved from 0.754 at 2–6 h, to 0.795 at 6–12 h, to 0.821 at 12–24 h. This echoes the findings of Roca et al. [18], where ROX measured at earlier time points had poorer sensitivity and specificity of < 70%. One possible explanation [20] for the better ROX index performance in later time windows is that patients may be relatively undifferentiated initially, but the additional time afforded them by HFNC therapy allows the underlying pace of COVID-19 disease to become evident, and allows for COVID-19 therapeutics (such as steroids or antiviral drugs) to exert their clinical effect. There were relatively few studies investigating the ROX index in the 0–2 h time window, and we were unable to perform an sAUC analysis. We are unable to comment on the overall diagnostic performance of the ROX index during this window, or to recommend a cut-off.

We suggest that at times closer to HFNC initiation, a cut-off that is highly specific for HFNC failure be used to identify patients at high risk of needing intubation. For these patients, early intubation should be considered. For 2–6 h post-HFNC initiation, we propose the cut-off of < 5.33 (80% specific); for 6–12 h post-HFNC initiation, we propose the cut-off of < 3.69 (80% specific).

On the other hand, at later time points, a cut-off that is highly sensitive for HFNC failure could be used to identify patients with higher likelihood of HFNC success. Such patients might benefit from continued treatment with HFNC. They might even be eligible for non-invasive monitoring with the ROX index given their high likelihood of HFNC success, as opposed to invasive monitoring with arterial blood gas sampling. For 12–24 h post-HFNC initiation, we propose the cut-off of > 6.07 (80% sensitive).

We have also conducted subgroup analyses that examined the ROX index in studies stratified by patient age, use of corticosteroids, and the study period. There were no significant differences in the diagnostic power of the ROX index when comparing among these subgroups, suggesting broad applicability of the ROX index regardless of older age, corticosteroid use, and across different years of the COVID-19 pandemic.

### Strengths and limitations

This is the first meta-analysis to estimate optimal cut-off values for the ROX index and evaluate its diagnostic accuracy at different time points and time windows, and to provide high sensitivity and high specificity cut-off values at each time point. This can allow clinicians to make informed choices about the ROX cut-off to use while the patient is undergoing HFNC therapy.

There are some limitations of this analysis. As with any systematic review, we are dependent on the availability of data from the constituent studies. First, publicly available data were only available in aggregate form, instead of individual patient data (IPD), which would be most ideal for estimating diagnostic test performance. This necessitated estimation of the sAUC from the aggregates, rather than directly calculating the AUC. We were thus also unable to analyse the distribution of the ROX index for patients in general, to determine if changes in ROX index within the same patient had any diagnostic value, or to examine the performance of the ROX index in detail for other subgroups (for example, vaccinated versus unvaccinated individuals, immunocompromised individuals, or individuals who received antiviral or immunomodulatory drugs). This lack of IPD necessitated the subgroup analysis to be performed based on study-level characteristics such as mean age and proportion of patients receiving corticosteroids. The lack of IPD, coupled with the sparsity of large studies with cut-off values that achieved 90% sensitivity/90% specificity, is a limitation to reliable calculation of estimated cut-off values for 90% sensitivity/90% specificity in subgroups such as time windows and nature of outcome. We also appreciate that sAUC estimated confidence intervals are relatively large, although this may be improved in the future as more data on HFNC use becomes available.

We are also aware that the absence of standardised reporting protocols for HFNC use can lead to sources of bias that are difficult to account for based on the extant literature. For example, few studies report at which point from onset of COVID-19 illness that the patient presented (which is potentially important as patient presenting very late or very early could have a different disease course [51–53]). Other sources of heterogeneity include the definition of HFNC failure, where some studies reported a composite outcome including mortality, on top of the need for intubation or mechanical ventilation. Another source of heterogeneity is the lack of reporting of intubation criteria. The variability in determining when intubation should be initiated would affect the performance of the ROX index. There was an overall lack of standardisation of the time horizon of outcomes in general. As more studies on HFNC and ROX are done, it may be worthwhile for consensus definitions of HFNC success and failure to be agreed on, and minimum reporting criteria for HFNC studies.

We are aware that modified versions of the ROX index exist, such as a modified ROX index [17] incorporating heart rate that was validated in a cohort of 145 patients. There is an overall paucity of data on modified ROX models, and the performance of these alternative indices may be better addressed in future meta-analyses as more data become available.

## Conclusion

The ROX index has a good diagnostic accuracy for HFNC failure in COVID-19 patients and performs the best at 6–12 h or later, post-initiation of HFNC. We suggest an optimal cut-off of 5.23 in general, but propose that healthcare providers also contextualise interpretation of the ROX index depending on the time from HFNC initiation. A higher specificity cut-off may be preferred closer to initiation to rule in HFNC failure, whereas a higher sensitivity cut-off could be used further from initiation to rule out HFNC failure and justify continuation of HFNC treatment.

### Supplementary Information

**Additional file 1**. Search strategy. **Fig. S1**: Risk-of-bias assessment using the quality assessment of prognostic accuracy studies (QUAPAS) tool.

## Data Availability

Data used for analysis can be made available upon reasonable request.
