# Supplementary material for: Performance of the ROX index in predicting high flow nasal cannula failure in COVID-19 patients: a systematic review and meta-analysis
Source: Crit Care. 2023 Aug 21;27:320. doi: 10.1186/s13054-023-04567-7 (PMC10441756; doi:10.1186/s13054-023-04567-7)
Supplement: Supplementary file 1 — Additional file 1. Search strategy. Fig. S1: Risk-of-bias assessment using the quality assessment of prognostic accuracy studies (QUAPAS) tool. [file 13054_2023_4567_MOESM1_ESM.docx]

**Supplementary Data 1: Search strategy**

**EMBASE**

| Concept | Search terms |
| --- | --- |
| ROX | (ROX or ROX-index or ROX* or "ROX index" or "respiratory rate-oxygenation" or ROXI OR ROX-I or "respiratory rate oxygenation"):ti,ab,kw |
| Covid-19 | **'covid 19'/exp OR 'coronavirus disease 2019'/exp OR 'coronavirus infection'/exp OR 'severe acute respiratory syndrome coronavirus 2'/exp OR '2019‐ncov':ti,ab OR '2019 ncov':ti,ab OR ncov:ti,ab OR 'covid‐19':ti,ab OR 'covid‐2019':ti,ab OR 'covid2019':ti,ab OR covid19:ti,ab OR 'sars‐cov‐2':ti,ab OR 'sars cov-2':ti,ab OR sarscov2:ti,ab OR 'sarscov‐2':ti,ab OR 'sars‐coronavirus‐2':ti,ab OR 'sars corona virus':ti,ab OR 'sars‐like coronavirus':ti,ab OR 'novel coronavirus':ti,ab OR 'novel corona virus':ti,ab OR covid*:ti,ab OR 'coronavirus 2':ti,ab OR 'coronavirus infection*':ti,ab OR 'coronavirus disease':ti,ab OR 'corona virus disease':ti,ab OR 'new coronavirus':ti,ab OR 'new corona virus':ti,ab OR 'new coronaviruses':ti,ab OR 'novel coronaviruses':ti,ab OR 'severe acute respiratory syndrome coronavirus‐2':ti,ab OR ((coronavirus:ti,ab OR ncov:ti,ab OR 'sars cov':ti,ab) AND (2019:ti,ab OR 19:ti,ab OR wuhan:ti,ab))** |

**Medline/PubMed**

| Concept | Search terms |
| --- | --- |
| ROX | (ROX OR ROX-index OR "ROX index" OR "respiratory rate-oxygenation" OR ROXI OR ROX-I OR "respiratory rate oxygenation"):ti,ab |
| Covid-19 | "SARS-CoV-2"[Mesh] OR "COVID-19"[Mesh] OR "Coronavirus Infections"[MeSH Terms] OR 2019‐nCoV[Title/Abstract] OR 2019 ncov[Title/Abstract] OR nCov[Title/Abstract] OR covid‐19[Title/Abstract] OR covid19[Title/Abstract] OR COVID-2019[Title/Abstract] OR COVID2019[Title/Abstract] OR sars‐cov‐2[Title/Abstract] OR "sars cov-2"[Title/Abstract] OR sarscov2[Title/Abstract] OR sarscov‐2[Title/Abstract] OR sars‐coronavirus‐2[Title/Abstract] OR sars corona virus[Title/Abstract] OR sars‐like coronavirus[Title/Abstract] OR novel coronavirus[Title/Abstract] OR novel corona virus[Title/Abstract] OR covid*[Title/Abstract] OR coronavirus 2[Title/Abstract] OR coronavirus infection*[Title/Abstract] OR coronavirus disease[Title/Abstract] OR corona virus disease[Title/Abstract] OR new coronavirus[Title/Abstract] OR new corona virus[Title/Abstract] OR new coronaviruses[Title/Abstract] OR novel coronaviruses[Title/Abstract] OR severe acute respiratory syndrome coronavirus‐2[Title/Abstract] OR ((coronavirus[Title/Abstract]  OR ncov[Title/Abstract]  OR sars-cov[Title/Abstract]) AND (2019[Title/Abstract] OR 19[Title/Abstract] OR Wuhan[Title/Abstract])) |

**Cochrane Central Register of Controlled Trials (CENTRAL)**

| Concept | Search terms |
| --- | --- |
| ROX | (ROX or ROX-index or ROX* or "ROX index" or "respiratory rate-oxygenation" or ROXI OR ROX-I or "respiratory rate oxygenation"):ti,ab,kw |
| Covid-19 | **'covid 19'/exp OR 'coronavirus disease 2019'/exp OR 'coronavirus infection'/exp OR 'severe acute respiratory syndrome coronavirus 2'/exp OR '2019‐ncov':ti,ab OR '2019 ncov':ti,ab OR ncov:ti,ab OR 'covid‐19':ti,ab OR 'covid‐2019':ti,ab OR 'covid2019':ti,ab OR covid19:ti,ab OR 'sars‐cov‐2':ti,ab OR 'sars cov-2':ti,ab OR sarscov2:ti,ab OR 'sarscov‐2':ti,ab OR 'sars‐coronavirus‐2':ti,ab OR 'sars corona virus':ti,ab OR 'sars‐like coronavirus':ti,ab OR 'novel coronavirus':ti,ab OR 'novel corona virus':ti,ab OR covid*:ti,ab OR 'coronavirus 2':ti,ab OR 'coronavirus infection*':ti,ab OR 'coronavirus disease':ti,ab OR 'corona virus disease':ti,ab OR 'new coronavirus':ti,ab OR 'new corona virus':ti,ab OR 'new coronaviruses':ti,ab OR 'novel coronaviruses':ti,ab OR 'severe acute respiratory syndrome coronavirus‐2':ti,ab OR ((coronavirus:ti,ab OR ncov:ti,ab OR 'sars cov':ti,ab) AND (2019:ti,ab OR 19:ti,ab OR wuhan:ti,ab))** |

**Supplementary Figure 1: Risk-of-bias assessment using the Quality Assessment of Prognostic Accuracy Studies (QUAPAS) tool**
